# Asymptomatic Patients with Severe Aortic Stenosis and the Impact of Intervention

**DOI:** 10.3390/jcdd8040035

**Published:** 2021-03-31

**Authors:** Mevlüt Çelik, Milan Milojevic, Andras P. Durko, Frans B. S. Oei, Edris A. F. Mahtab, Ad J. J. C. Bogers

**Affiliations:** Department of Cardiothoracic Surgery, Erasmus University Medical Center, 3015 GD Rotterdam, The Netherlands; m.celik@erasmusmc.nl (M.Ç.); mln.milojevic@gmail.com (M.M.); andras.durko@gmail.com (A.P.D.); f.oei@erasmusmc.nl (F.B.S.O.); a.j.j.c.bogers@erasmusmc.nl (A.J.J.C.B.)

**Keywords:** aortic valve replacement, aortic stenosis, asymptomatic, early surgery, watchful waiting

## Abstract

Objectives the exact timing of aortic valve replacement (AVR) in asymptomatic patients with severe aortic stenosis (AS) remains a matter of debate. Therefore, we described the natural history of asymptomatic patients with severe AS, and the effect of AVR on long-term survival. **Methods:** Asymptomatic patients who were found to have severe AS between June 2006 and May 2009 were included. Severe aortic stenosis was defined as peak aortic jet velocity Vmax ≥ 4.0 m/s or aortic valve area (AVA) ≤ 1 cm^2^. Development of symptoms, the incidence of AVR, and all-cause mortality were assessed. **Results:** A total of 59 asymptomatic patients with severe AS were followed, with a mean follow-up of 8.9 ± 0.4 years. A total of 51 (86.4%) patients developed AS related symptoms, and subsequently 46 patients underwent AVR. The mean 1-year, 2-year, 5-year, and 10-year overall survival rates were higher in patients receiving AVR compared to those who did not undergo AVR during follow-up (100%, 93.5%, 89.1%, and 69.4%, versus 92.3%, 84.6%, 65.8%, and 28.2%, respectively; *p* < 0.001). Asymptomatic patients with severe AS receiving AVR during follow-up showed an incremental benefit in survival of up to 31.9 months compared to conservatively managed patients (*p* = 0.002). **Conclusions:** The majority of asymptomatic patients turn symptomatic during follow-up. AVR during follow-up is associated with better survival in asymptomatic severe AS patients.

## 1. Introduction

Aortic stenosis (AS) is the most common valvular heart disease, with a prevalence of approximately 5% in adults above the age of 65 years [1]. The prevalence is expected to grow exponentially in the coming decades due to the aging population in developed countries [2]. Patients with symptomatic severe AS currently hold a class IB recommendation for surgical aortic valve replacement (SAVR) treatment, due to the dismal prognosis once symptoms are present [3,4]. Yet, up to 50% of the patients with severe AS report no symptoms at initial diagnosis [5].

Due to the low risk of sudden cardiac death, which is believed to be approximately 1%, a conservative approach is currently the treatment of choice in the asymptomatic population. New evidence challenges this belief, and the incidence of sudden death might be higher than previously expected [6]. In addition, the majority of these patients develop AS related symptoms and require intervention within the first 2 years of follow-up [7]. In the present study, we aimed to study the natural history of a cohort of consecutive asymptomatic patients with severe AS, and to evaluate the implications of aortic valve intervention (AVR) on long-term survival.

## 2. Methods

### 2.1. Patient Population

This retrospectively analyzed, prospective multicenter study enrolled asymptomatic adult (≥18 years) patients diagnosed with severe AS at seven Cardiology clinics in the Rotterdam area between June 2006 and May 2009. Patients were deemed asymptomatic if they had no cardiac symptoms at baseline visit (angina, shortness of breath, or syncope). In accordance with the European Society of Cardiology and American College of Cardiology/American Heart Association Guidelines for the Management of Patients With Valvular Heart Disease, severe AS was defined as aortic jet maximal velocity Vmax ≥ 4.0 m/s or aortic valve area (AVA) ≤ 1 cm^2^ [8,9]. Patients had a normal left ventricular ejection fraction (≥50%). After inclusion in the present cohort, asymptomatic patients were invited for exercise testing at baseline. A positive exercise test was defined according to the ACC/AHA guidelines [10]. The study was approved by the medical ethics committee of the Erasmus University Medical Center, and patient informed consent was waived. All authors vouch for the validity of the data and adherence to the protocol.

### 2.2. Endpoints and Definitions

The primary endpoint was all-cause mortality. The secondary endpoints were the development of AS related symptoms and the need for AVR with either SAVR or transcatheter aortic valve implantation (TAVI). SAVR within 24 h of establishing the indication was classified as urgent.

### 2.3. Statistical Analysis

Discrete variables are presented as numbers, percentages, or proportions. Continuous variables are presented as means ± standard deviation, and presented as median with the interquartile range (IQR) if there was evidence of skewed data according to the Kolmogorov–Smirnov test. Discrete variables were compared with either the Chi Square test or the Fisher exact test, where appropriate. Continuous variables were compared with either the two-sample t-test or Wilcoxon rank-sum test, where appropriate.

Cumulative incidences were assessed using Kaplan–Meier curves to estimate the probability of: (i) symptom development, (ii) AVR, (iii) all-cause mortality in the overall cohort, and (iv) all-cause mortality in patients separated by whether they underwent AVR during follow-up. The incidence of AVR during follow-up was calculated and expressed as the number of AVRs per 1000 patient-years.

Predictors of (i) all-cause mortality and (ii) AVR were identified by a Cox proportional hazards model. Significant variables on univariable analyses were included in a multivariable Cox proportional hazards model. Furthermore, the restricted mean survival time at 10-years of follow-up was calculated to substantiate the overall treatment effect. Two-sided *p*-values < 0.05 were considered to be statistically significant. Data analyses were performed using SPSS 25.0 (SPSS Inc., Chicago, IL, USA) and R software, version 3.4 (R Foundation, Vienna, Austria).

## 3. Results

### 3.1. Baseline Characteristics

The final study population consisted of 59 asymptomatic patients with severe AS (Supplementary Materials, Figure S1). The mean age of the patients was 68.2 ± 10.7 years. Patients receiving AVR during follow-up were younger compared to patients with a conservative approach, 66.5 ± 10.6 versus 74.1 ± 8.9; *p* = 0.022, respectively. Asymptomatic patients with AVR during follow-up had a trend toward being female (30.4% versus 7.7%, *p* = 0.096) and had less diabetes mellitus (13.0% versus 46.2%, *p* = 0.009). No difference in baseline severity of AS was noted, based on AVA (0.85 ± 0.27 versus 0.80 ± 0.30, *p* = 0.536) and Vmax (4.23 ± 0.68 versus 4.28 ± 0.70, *p* = 0.823). Further baseline characteristics for the overall cohort and patients undergoing AVR and no AVR during follow-up are shown in Table 1.

### 3.2. Natural Course of Asymptomatic Severe Stenosis

**Figure 1:**
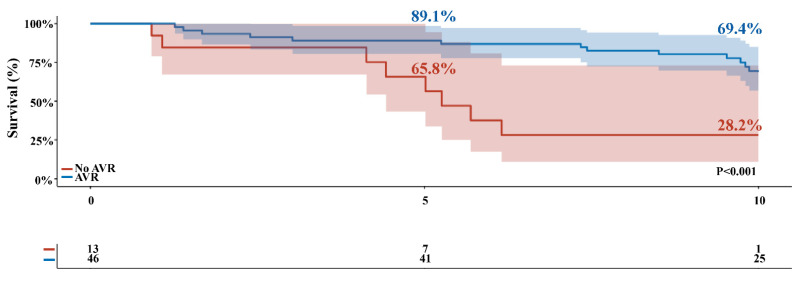
Survival during follow-up. Actual survival of asymptomatic patients according to having received AVR during follow-up. Blue line represents patients who underwent AVR during follow-up. Red line represents patients who did not undergo AVR during follow-up. Shaded region represents the 95% confidence interval. AVR, aortic valve replacement.

Forty-seven out of the 59 patients underwent an exercise stress test at baseline. Of these 47 patients, 15 (32%) tested positive and 32 (68%) patients tested negative. The other twelve patients were unable to undergo an exercise stress test. Nearly half of the patients had their symptoms unmasked by baseline exercise test or eventually developed symptoms within the first year after initial diagnosis (*n* = 26; 44%), but the vast majority of patients had symptoms (*n* = 51/59, 86.4%) before AVR or death (Figure 1). Mean time to symptom onset was 2.6 ± 0.4 years. During follow-up, 46 patients required AVR, of whom 11 (24.4%) had a positive exercise test at baseline. Three patients underwent TAVI. Eight patients died before undergoing AVR. Cumulative incidence of AVR was 13.6% and 91.4% at 1-year and 10-years, respectively (Figure 1). The linearized incidence rate of AVR was 95.5 per 1000 patient-years. Baseline characteristics of patients who did not undergo AVR according to survival status is shown in Table S1.

### 3.3. Survival

**Figure 2:**
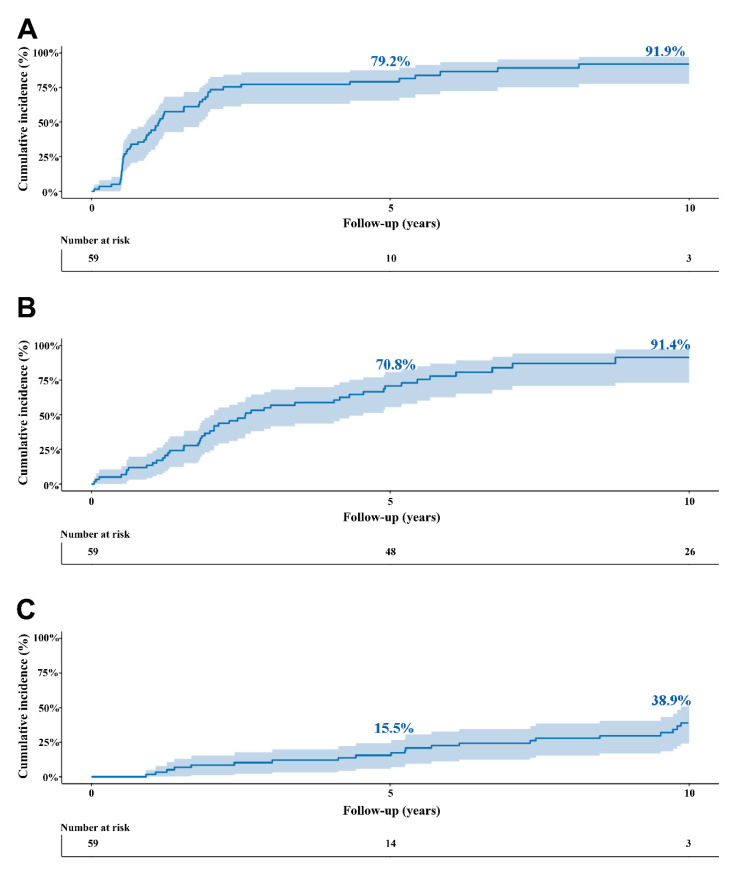
Cumulative incidence rates in the overall cohort. (**A**) symptom development, (**B**) aortic valve replacement (either surgical or transcatheter), and (**C**) all-cause mortality. Shaded region represents the 95% confidence interval.

During the mean follow-up time of 8.9 ± 0.4 years, 35 patients (59.3%) died. Early (30-day) mortality after AVR occurred in 0 patients. The incidence of all-cause mortality was 38.9% at 10-years in the overall cohort (Figure 2C). The mean 1-year, 2-year, 5-year, and 10-year overall survival rates was higher in patients receiving AVR compared to conservatively managed patients (100%, 93.5%, 89.1%, and 69.4% versus 92.3%, 84.6%, 65.8%, and 28.2% respectively; *p* < 0.001) (Figure 2). Patients receiving AVR during follow-up had a 31.9-month survival benefit over 10 years of follow-up (95% confidence interval (CI): 13.27–58.44, *p* = 0.002) compared to conservatively managed patients (Table 2).

### 3.4. Predictors of Outcome

In univariable analyses, being older (HR 1.11, 95% CI 1.06–1.17), having higher NT-proBNP levels (HR 1.002, 95% CI 1.001–1.004), having diabetes mellitus (HR = 4.57, 95% CI 1.91–10.96), atrial fibrillation (HR 4.98, 95% CI 1.40–17.72), and AVR during follow-up (HR 0.24, 95% CI 0.10–0.58) were predictors of all-cause mortality (Table 3). Age remained the only predictor after multivariable analysis (HR 1.08, 1.01–1.16, *p* = 0.026). Univariate predictors of AVR in asymptomatic patients are shown in Table S2.

## 4. Discussion

This study describes the natural history of asymptomatic patients with severe AS and the impact of intervention in this patient population. We found that (i) the majority of the patients eventually develop AS related symptoms, (ii) subsequently requiring AVR, and that (iii) patients who received AVR had a survival benefit of close to three years compared to conservatively managed patients.

Adriana C. Gittenberger-de Groot and her team have performed an extensive number of indispensable studies on the spectrum of aortic valvular disease over the past decades, including histopathological, anatomical, and developmental studies on animal, as well as human, tissue [10].

The majority of asymptomatic patients developed symptoms within the first 3 years after initial diagnosis [11], with up to 86.4% at 10-years in our cohort. The asymptomatic patient might be “falsely” labelled as asymptomatic. In our cohort 79.7% underwent exercise stress testing at baseline, of whom 31.9% of the patients had a positive test. Abnormal exercise test is associated with impaired 2-year event-free survival [12,13,14], and is a clear indication of AVR [3,4]; especially in elderly patients who are subconsciously adapting their exercise to their tolerance and underrepresent their symptoms. It is still concerning that relatively few asymptomatic patients in practice undergo routine stress testing [15]. Several difficulties with exercise testing exist in the elderly population, including (1) its lower predictive value compared to a younger population, (2) limited exercise capability in the elderly due to non-cardiac conditions limiting mobility, and (3) the differences in exercise protocol and definition of an abnormal exercise test [16,17]. The relevancy and accuracy of exercise testing is therefore still a debated topic.

The majority of the asymptomatic population with severe AS who develop symptoms underwent AVR (91.4%). Our rate was higher in comparison with earlier reports, wherein approximately 57% of the patients underwent AVR at 10-years of follow-up [11]. This discrepancy could be caused by the recommendation of the physician. The current asymptomatic patient with severe AS does not have a formal indication for intervention, unless the patient has (1) depressed LVEF, or (2) is undergoing concomitant cardiac surgery [3,4]. Yet, it is expected that the degree of AS will gradually increase, and the initially asymptomatic patient eventually will develop symptoms due to disease progression, and subsequently requiring the guideline recommended AVR. The upfront gain obtained by delaying surgery might not outweigh the risk of AVR being delayed with conservative treatment. This is especially the case in patients who are older, and subsequently have increased operative risk [18]. In those patients the long-term hemodynamic consequences might outweigh the positive outcomes of an early interventional strategy [19,20].

Asymptomatic patients with severe AS undergoing AVR during follow-up had better survival compared to conservatively managed patients. In the first randomized controlled trial, a total of 145 asymptomatic patients with very severe AS were randomized to early surgery (*n* = 73) and conservative care (*n* = 72) [21]. Early surgery resulted in improved survival at 8-years of follow-up compared to patients treated with a conservative approach (90% versus 74%, *p* = 0.003, respectively). However, this study only provides a perspective on patients with very severe AS. Initial data in asymptomatic patients with severe AS on all-cause mortality at 5-year from the CURRENT AS registry indicate a survival benefit for patients undergoing surgery within 3-months compared to conservative treatment, 26.4% vs. 15.4%; *p* = 0.009, respectively [20]. While a pre-emptive strategy seems superior in those with (very) severe AS [21], the exact timing and benefit of AVR in asymptomatic patients with severe AS remains to be defined. The exact timing may be refined with improvements in imaging modalities. Measuring the aortic valve calcium score through computed tomography has emerged as a strategy to assess the severity and progression of aortic stenosis, especially in asymptomatic patients with echocardiographic discordance [22]. With the advent of TAVI, conservative treatment is a solution that almost nobody still considers. The role of minimally invasive techniques and imaging modalities in the asymptomatic cohort with severe AS will need to be substantiated in the future (NCT03094143 and NCT03042104, Table S3).

## 5. Limitations

Several limitations need to be acknowledged. First, this is an retrospective study, with the inherent shortcomings. Second, the number of patients and subsequent events was relatively low, with shortcomings related to overfitting of multivariable analyses. Given the fact that patients were not randomized into early surgical management and conservative treatment, potential selection bias cannot be eliminated, wherein an older patient was less likely to undergo AVR, as the indication for treatment was left to the discretion of the treating physician.

## 6. Conclusions

The vast majority of asymptomatic patients with severe AS develop symptoms during follow-up and subsequently require intervention. Intervention during follow-up is associated with better long-term survival, and early intervention is likely to improve survival. Close clinical follow-up is warranted for all patients, and pre-emptive elective aortic valve procedures may be considered in selected elderly patients at low procedural risk. Further results from the currently ongoing clinical trials will give us more insight into the role of early intervention in asymptomatic patients with severe AS.

## Figures and Tables

**Table 1:** Baseline characteristics of the asymptomatic population.

	All (*n* = 59)	Conservative Treatment (*n* = 13)	AVR (*n* = 46)	*p*-Value
Age (years)	68.8 ± 10.6	74.1 ± 8.9	66.5 ± 10.6	0.022
Female	15 (25.4)	1 (7.7)	14 (30.4)	0.096
BMI	27.1 ± 3.7	27.5 ± 3.9	26.9 ± 3.7	0.661
BSA	1.93 ± 0.20	2.00 ± 0.12	1.91 ± 0.21	0.226
Previous CABG	2 (3.4)	0	2 (4.3)	0.444
Smoking	42 (71.2)	10 (76.9)	32 (69.6)	0.605
Atrial fibrillation	4 (7.0)	2 (15.4)	2 (4.5)	0.179
Carotid disease	1 (1.7)	1 (7.7)	0	0.058
Coronary artery disease	4 (6.8)	0	4 (8.7)	0.271
COPD	6 (10.2)	2 (15.4)	4 (8.7)	0.481
Diabetes	12 (20.3)	6 (46.2)	6 (13.0)	0.009
Hyperlipidemia	29 (49.2)	8 (61.5)	21 (45.7)	0.312
Hypertension	29 (49.2)	5 (38.5)	24 (52.2)	0.383
Myocardial infarction	4 (6.8)	0	4 (8.7)	0.271
Peripheral arterial disease	5 (8.5)	0	5 (10.9)	0.214
Stroke	12 (20.3)	3 (23.1)	9 (19.6)	0.781
NT-proBNP (pmol/l)	32.0 (18.0–97.0)	33.0 (12.8–149.3)	32.0 (18.0–89.0)	0.976
Baseline positive stress test	15 (25.4)	4 (30.8)	11 (24.4)	0.646
Logistic EuroSCORE	4.0 (2.1–6.9)	4.7 (3.2–8.1)	3.9 (2.1–5.5)	0.485
STS score	3.8 (2.0–6.0)	5.2 (2.2–8.6)	3.6 (2.0–5.0)	0.403
No medication	13 (22.0)	2 (15.4)	11 (23.9)	0.512
Diuretics	11 (18.6)	3 (23.1)	8 (17.4)	0.642
Ace Inhibitor	14 (23.7)	4 (30.8)	10 (21.7)	0.499
A2 antagonist	11 (18.6)	5 (38.5)	6 (13.0)	0.038
B blocker	15 (25.4)	1 (7.7)	14 (30.4)	0.096
Calcium antagonist	8 (13.6)	2 (15.4)	6 (13.0)	0.828
Digoxine	4 (6.8)	0	4 (8.7)	0.271
**Echocardiographic Parameters**				
Vmax (m/s)	4.24 ± 0.68	4.28 ± 0.70	4.23 ± 0.68	0.823
AVA (cm^2^)	0.85 ± 0.28	0.80 ± 0.30	0.85 ± 0.27	0.536
iAVA (cm^2^/m^2^)	0.44 ± 0.15	0.41 ± 0.16	0.44 ± 0.14	0.423
MAG (mmHg)	42.8 ± 15.0	44.3 ± 17.4	42.3 ± 14.4	0.684
PAG (mmHg)	73.2 ± 23.6	75.3 ± 24.1	72.6 ± 23.7	0.720
AR grade I/II	29 (50.0)	6 (46.2)	23 (51.1)	0.753
MR grade I/II	12 (20.7)	4 (30.8)	8 (17.8)	0.308
LVEF	62.5 ± 5.9	61.1 ± 5.9	62.7 ± 5.7	0.374
LF/LG AS (%)	5 (8.5)	0	5 (10.9)	0.214
LVH (%)	14 (25.5)	2 (16.7)	12 (27.9)	0.429
TAPSE (mm)	25.1 ± 3.7	23.6 ± 2.8	25.5 ± 3.9	0.104
LVEDD (mm)	49.0 ± 6.0	49.6 ± 5.1	25.5 ± 3.9	0.687
LVESD (mm)	31.4 ± 6.2	30.3 ± 5.7	31.7 ± 6.4	0.466
LVFS (%)	36.1 ± 8.8	38.6 ± 11.0	35.4 ± 8.1	0.248
LA (mm)	41.3 ± 6.4	42.2 ± 6.8	41.0 ± 6.3	0.563
IVSd (mm)	12.6 ± 2.7	12.5 ± 2.0	12.6 ± 2.9	0.834
IVCd (mm)	17.4 ± 3.6	16.4 ± 2.7	17.7 ± 3.8	0.252
PWd (mm)	10.8 ± 2.0	11.5 ± 1.7	10.7 ± 2.1	0.161
E′ (cm/s)	79.5 ± 23.6	69.1 ± 29.9	82.0 ± 21.4	0.103
A′ (cm/s)	89.9 ± 37.2	104.2 ± 59.9	86.0 ± 27.9	0.134
E′A′ ratio	1.0 ± 0.57	0.8 ± 0.5	1.1 ± 0.6	0.120
LVET (ms)	322.1 ± 32.2	312.6 ± 43.9	324.8 ± 28.4	0.296
DT (ms)	239.4 ± 63.3	217.5 ± 52.6	245.3 ± 65.2	0.198

Values are presented as mean ± SD or *n* (%). A′, peak velocity of diastolic mitral annular motion; AR, aortic regurgitation; AVA, aortic valve area; BMI, body mass index; BSA, body surface area; COPD, chronic obstructive pulmonary disease; DT, deceleration time; iAVA, indexed aortic valve area; E′, peak velocity of early diastolic mitral annular motion; E′A′ ratio, ratio of E′ to A′; IVCd, inferior vena cava dimension; IVSd, interventricular septum dimension; LA, left atrium; LF/LG AS, low-flow/low-grade AS; LVEDD, left ventricular end diastolic diameter; LVEF, left ventricular ejection fraction; LVESD, left ventricular end systolic diameter; LVET, left ventricular ejection time; LVFS, left ventricular fractional shortening; LVH, left ventricular hypertrophy; MAG, mean aortic gradient; MR, mitral regurgitation; PAD, peripheral arterial disease; PAG, peak aortic gradient; PWd, posterior wall dimension; TAPSE, tricuspid annular plane systolic excursion; Vmax, maximal velocity.

**Table 2:** Between-group differences in mortality among treatment strategies (conservative versus AVR).

Overall Cohort			
Restricted Mean Survival Time at 10 Years		95% CI	*p*-Value
Difference—months	31.85	13.27–58.44	0.002
Ratio	1.51	1.11–2.05	0.008
Ratio of restricted mean time lost	0.28	0.13–0.60	0.001

AVR, aortic valve replacement; CI, confidence interval.

**Table 3:** Predictors of all-cause mortality in the overall cohort during follow-up.

	Univariable HR (95% CI), *p*-Value	Multivariable HR (95% CI), *p*-Value
Age	1.11 (1.06–1.17), *p* < 0.001	1.08 (1.01–1.16), *p* = 0.026
Gender (female)	0.43 (0.16–1.37), *p* = 0.125	
Atrial fibrillation	4.98 (1.40–17.72), *p* = 0.013	3.10 (0.68–14.26), *p* = 0.146
Coronary artery disease	0.62 (0.08–4.59), *p* = 0.639	
COPD	1.60 (0.48–5.39), *p* = 0.446	
Diabetes mellitus	4.57 (1.91–10.96), *p* < 0.001	2.36 (0.87–6.44), *p* = 0.094
Hyperlipidemia	1.65 (0.72–3.78), *p* = 0.234	
Hypertension	1.50 (0.66–3.37), *p* = 0.332	
Myocardial infarction	1.14 (0.27–4.86), *p* = 0.862	
Peripheral arterial disease	1.58 (0.46–5.34), *p* = 0.466	
Stroke	2.11 (0.90–4.92), *p* = 0.086	
Exercise test (positive)	0.74 (0.27–1.98) *p* = 0.543	
NT-proBNP	1.002 (1.001–1.004) *p* < 0.001	1.002 (1.00–1.003), *p* = 0.053
STS score	1.06 (0.99–1.14), *p* = 0.098	
Logistic EuroSCORE	1.22 (1.09–1.35), *p* < 0.001	0.98 (0.81–1.18), *p* = 0.830
AVR	0.24 (0.10–0.58) *p* = 0.002	1.17 (0.31–4.36), *p* = 0.820
LVEF	0.96 (0.90–1.04), *p* = 0.315	
Vmax	0.95 (0.53–1.70), *p* = 0.851	
AVA	0.19 (0.03–1.11), *p* = 0.065	
iAVA	0.05 (0.00–1.141), *p* = 0.078	
MAG	1.01 (0.99–1.03), *p* = 0.460	
PAG	1.00 (0.98–1.10), *p* = 0.817	

AVA, aortic valve area; AVR, aortic valve replacement; COPD: chronic obstructive pulmonary disease; iAVA, indexed aortic valve area; LVEF, left ventricular ejection fraction; MAG, mean aortic gradient; PAG, peak aortic gradient; STS, Society of Thoracic Surgery; Vmax, maximal jet velocity.

## Data Availability

Not applicable.

## Supplementary Materials

The following are available online at https://www.mdpi.com/article/10.3390/jcdd8040035/s1, Figure S1. Flowchart of the patients during follow-up. A total of 59 asymptomatic patients with severe AS were included. Total of 51 patients did develop symptoms and 8 did not. Of whom who did develop symptoms underwent 42 AVR, and 15 patients died after AVR. Of whom who did not develop symptoms 3 underwent AVR, and 1 died after AVR. Five patients died after symptoms without undergoing AVR. Four patients died with no symptoms without undergoing AVR.

## Abbreviations

A′	peak velocity of diastolic mitral annular motion
AR	aortic regurgitation
AS	aortic stenosis
AVA	aortic valve area
AVR	aortic valve replacement
BMI	body mass index
BSA	body surface area
CI	confidence interval
COPD	chronic obstructive pulmonary disease
E′	peak velocity of early diastolic mitral annular motion
E′A′ ratio	ratio of E′ to A
EuroSCORE	European system for cardiac operative risk evaluation
HR	hazard ratio
iAVA	indexed aortic valve area
IVCd	inferior vena cava dimension
IVSd	interventricular septum dimension
LA	left atrium
LF/LG AS	low-flow/low-grade AS
LVEDD	left ventricular end diastolic diameter
LVEF	left ventricular ejection fraction
LVET	left ventricular ejection time
LVESD	left ventricular end systolic diameter
LVFS	left ventricular fractional shortening
LVH	left ventricular hypertrophy
MAG	mean aortic gradient
MR	mitral regurgitation
PAD	peripheral arterial disease
PAG	peak aortic gradient
PWd	posterior wall dimension
RCT	randomized controlled trial
SAVR	surgical aortic valve replacement
STS	society of thoracic surgery
TAPSE	tricuspid annular plane systolic excursion
TAVI	transcatheter aortic valve implantation
Vmax	maximal velocity
