# Supplementary material for: Asymptomatic Patients with Severe Aortic Stenosis and the Impact of Intervention
Source: J Cardiovasc Dev Dis. 2021 Mar 31;8(4):35. doi: 10.3390/jcdd8040035 (PMC8066001; doi:10.3390/jcdd8040035)

Table 1. – Baseline characteristics of patients who did not undergo AVR during follow-up.

	No AVR during follow-up (n=13)	Died (n=8)	Alive (n=5)	P-value
**Age (years)**	74.1 ± 8.9	78.0 ± 7.7	67.8 ± 7.6	0.039
**Female**	1 (7.7)	0	1 (20.0)	0.188
**BMI**	27.5 ± 3.9	25.6 ± 2.0	30.4 ± 4.7	0.027
**BSA**	2.00 ± 0.12	2.0 ± 0.1	2.0 ± 0.1	0.572
**Previous CABG**	0	0	0	>0.999
**Smoking**	10 (76.9)	7 (87.5)	3 (60.0)	0.252
**Atrial fibrillation**	2 (15.4)	1 (12.5)	1 (20.0)	0.715
**Carotid disease**	1 (7.7)	1 (12.5)	0	0.411
**Coronary artery disease**	0	0	0	>0.999
**COPD**	2 (15.4)	1 (12.5)	1 (20.0)	0.715
**Diabetes**	6 (46.2)	5 (62.5)	1 (20.0)	0.135
**Hyperlipidemia**	8 (61.5)	6 (75.0)	2 (40.0)	0.207
**Hypertension**	5 (38.5)	4 (50.0)	1 (20.0)	0.279
**Myocardial infarction**	0	0	0	>0.999
**Peripheral arterial disease**	0	0	0	>0.999
**Stroke**	3 (23.1)	2 (25.0)	1 (20.0)	0.835
**NT-proBNP (pmol/l)**	33.0 (12.8-149.3)	100.5 (19.0-971.5)	35.0 (31.0-50.0)	0.425
**Baseline positive stress test**	4 (30.8)	3 (37.5)	1 (20.0)	0.506
**Logistic EuroSCORE**	4.7 (3.2-8.1)	7.1 (4.4-10.5)	3.0 (2.1-5.7)	0.121
**STS score**	5.2 (2.2-8.6)	5.8 (3.4-9.6)	3.4 (2.4-6.0)	0.845
**No medication**	2 (15.4)	1 (12.5)	1 (20.0)	0.715
**Diuretics**	3 (23.1)	2 (25.0)	2 (40.0)	0.835
**Ace Inhibitor**	4 (30.8)	3 (37.5)	1 (20.0)	0.506
**A2 antagonist**	5 (38.5)	5 (62.5)	0	0.024
**B blocker**	1 (7.7)	1 (12.5)	0	0.411
**Calcium antagonist**	2 (15.4)	2 (25.0)	0	0.224
**Digoxine**	0	0	0	>0.999
		**Echocardiographic parameters**		
**Vmax (m/s)**	4.28 ± 0.70	4.28 ± 0.85	4.28 ± 0.45	>0.999
**AVA (cm^2)**	0.80 ± 0.30	0.70 ± 0.26	0.95 ± 0.32	0.149
**iAVA (cm^2/m^2)**	0.41 ± 0.16	0.36 ± 0.14	0.48 ± 0.17	0.190
**MAG (mmHg)**	44.3 ± 17.4	47.1 ± 21.9	39.7 ± 4.8	0.476
**PAG (mmHg)**	75.3 ± 24.1	76.1 ± 29.4	74.0 ± 15.4	0.885
**AR grade I/II**	6 (46.2)	4 (50.0)	2 (40.0)	0.725
**MR grade I/II**	4 (30.8)	3 (37.5)	1 (20.0)	0.506
**LVEF**	61.1 ± 5.9	59.9 ± 6.4	63.1 ± 5.1	0.376
**LF/LG AS (%)**	0	0	0	>0.999
**LVH (%)**	2 (16.7)	2 (100.0)	0	0.190
**TAPSE (mm)**	23.6 ± 2.8	23.0 ± 2.6	24.6 ± 3.3	0.345
**LVEDD (mm)**	49.6 ± 5.1	48.4 ± 6.1	51.6 ± 1.8	0.288
**LVESD (mm)**	30.3 ± 5.7	32.7 ± 4.7	26.4 ± 5.2	0.044
**LVFS (%)**	38.6 ± 11.0	32.2 ± 6.6	48.9 ± 9.0	0.002
**LA (mm)**	42.2 ± 6.8	44.9 ± 6.4	37.9 ± 5.4	0.072
**IVSd (mm)**	12.5 ± 2.0	13.0 ± 2.1	11.6 ± 1.5	0.231
**IVCd (mm)**	16.4 ± 2.7	15.9 ± 3.1	16.5 ± 5.0	0.542
**PWd (mm)**	11.5 ± 1.7	11.3 ± 1.1	12.0 ± 2.4	0.448
**E’ (cm/s)**	69.1 ± 29.9	69.1 ± 33.9	69.0 ± 21.6	0.997
**A’ (cm/s)**	104.2 ± 59.9	88.0 ± 33.1	136.6 ± 92.2	0.198
**E’A’ ratio**	0.8 ± 0.5	0.9 ± 0.6	0.6 ± 0.4	0.377
**LVET (ms)**	312.6 ± 43.9	321 ± 29.5	300 ± 63.0	0.491
**DT (ms)**	217.5 ± 52.6	228.4 ± 55.7	188.3 ± 34.8	0.283

values are presented as mean ± SD or n (%)

***A'**, peak velocity of diastolic mitral annular motion; **AR**, aortic regurgitation; **AVA**, aortic valve area; **BMI**, body mass index; **BSA**, body surface area; **COPD**, chronich obstructive pulmonary disease; **DT**, deceleration time; **iAVA** , indexed aortic valve area; **E'**, peak velocity of early diastolic mitral annular motion; **E'A' ratio**, ratio of E' to A'; **IVCd**, inferior vena cava dimension; **IVSd**, interventricular septum dimension; **LA**, left atrium; **LF/LG AS**, low-flow/low-grade AS; **LVEDD**, left ventricular end diastolic diameter; **LVEF**, left ventricular ejection fraction; **LVESD**, left ventricular end systolic diameter; **LVET**, left ventricular ejection time; **LVFS**, left ventricular fractional shortening; **LVH**, left ventricular hypertrophy; **MAG** , mean aortic gradient; **MR**, mitral regurgitation; **PAD**, peripheral arterial disease; **PAG**, peak aortic gradient; **PWd**, posterior wall dimension, **TAPSE**, tricuspid annular plane systolic excursion; **Vmax**, maximal velocity*

Table S2. Predictors of AVR in asymptomatic patients during follow-up

	Univariable HR (95% CI), P value
**Age**	0.98 (0.95-1.01), p=0.125
**Gender (female)**	1.52 (0.80-2.90), p=0.200
**LVEF**	1.01 (0.97-1.06), p=0.556
**Atrial fibrillation**	0.51 (0.12-2.15), p=0.362
**Coronary artery disease**	1.15 (0.41-3.27), p=0.790
**COPD**	0.88 (0.31-2.46) p=0.806
**Diabetes mellitus**	0.57 (0.24-1.36) p=0.206
**Hyperlipidemia**	0.68 (0.38-1.23) p=0.205
**Hypertension**	1.25 (0.70-2.23) p=0.457
**Myocardial infarction**	2.07 (0.73-5.85), p=0.172
**Peripheral arterial disease**	1.33 (0.52-3.38), p=0.553
**Stroke**	0.80 (0.39-1.67), p=0.557
**Exercise test (positive)**	1.24 (0.62-2.48), p=0.540
**NT-proBNP**	1.00 (1.00-1.00) p=0.182
**STS**	1.00 (0.92-1.08), p=0.938
**Logistic EuroSCORE**	0.97 (0.88-1.07), p=0.593
**LVEF**	1.01 (0.97-1.06), p=0.556
**Vmax**	1.52 (0.99-2.32), p=0.056
**AVA**	0.51 (0.17-1.50), p=0.219
**iAVA**	0.38 (0.05-2.85), p=0.344
**MAG**	1.02 (1.00-1.03), p=0.093
**PAG**	1.01 (1.00-1.02), p=0.100

AVA, *aortic valve area;* ***AVR***, *aortic valve replacement;* ***COPD***: *chronic obstructive pulmonary disease;* ***iAVA***, *indexed aortic valve area;* ***LVEF***, *left ventricular ejection fraction;* ***MAG***, *mean aortic gradient;* ***PAG***, *peak aortic gradient;* ***STS***, *Society of Thoracic Surgery;* ***Vmax***, *maximal jet velocity.*

Table S3. Current on-going RCTs in the asymptomatic population

Name study	Source	SAVR or TAVR
RECOVERY	(https://clinicaltrials.gov/ct2/show/NCT01161732)	SAVR
AVATAR	(https://clinicaltrials.gov/ct2/show/NCT02436655)	SAVR
ESTIMATE	(https://clinicaltrials.gov/ct2/show/NCT02627391)	SAVR
EVoLVeD	(https://clinicaltrials.gov/ct2/show/NCT03094143)	SAVR/TAVR
EARLY TAVR	(https://clinicaltrials.gov/ct2/show/NCT03042104)	TAVR

Figure S1. Flowchart of the patients during follow-up

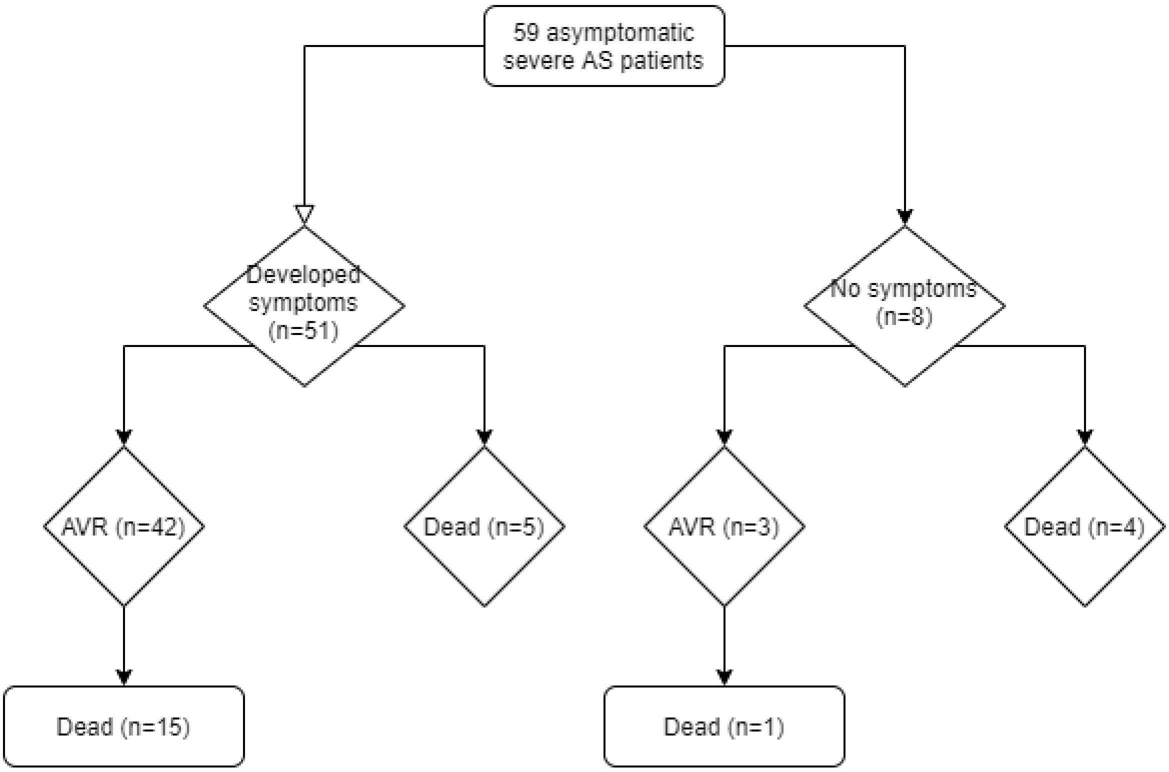

Supplement: Supplementary file 1 [file jcdd-08-00035-s001.pdf]
